# Evaluating left ventricular function and myocardial fibrosis in hypertrophic obstructive cardiomyopathy with corrected myocardial work

**DOI:** 10.1186/s44156-026-00113-7

**Published:** 2026-04-03

**Authors:** Xin Zhang, Lingyun Fang, Yuji Xie, Yuman Li, He Li, Manwei Liu, Chenchen Sun, Yun Yang, Ruize Zhang, Jia Xu, Jing Wang, Li Zhang, Qing Lv, Shu Chen, Wenqian Wu, Mingxing Xie

**Affiliations:** 1 Department of Ultrasound Medicine, Union Hospital, Tongji Medical College, Huazhong University of Science and Technology, 1277 Jiefang Avenue, Wuhan, 430022 China; 2 Hubei Province Clinical Research Center for Medical Imaging, Wuhan, China; 3 Hubei Province Key Laboratory of Molecular Imaging, Wuhan, China; 4 Department of Cardiovascular Surgery, Union Hospital, Tongji Medical College, Huazhong University of Science and Technology, Wuhan, China

**Keywords:** Myocardial work, Hypertrophic cardiomyopathy, Septal myectomy, Left ventricular function, Myocardial fibrosis

## Abstract

**Background:**

Echocardiographic Myocardial work (MW) has potential value in hypertrophic obstructive cardiomyopathy (HOCM). This study aimed to utilize corrected MW indices to characterize left ventricular (LV) myocardial mechanical remodeling and evaluate the extent of myocardial fibrosis (MF) in patients with HOCM.

**Methods:**

We prospectively studied 41 patients with HOCM undergoing septal myectomy (SM). 21 patients underwent intraoperative invasive pressure measurement to validate the noninvasive left ventricular systolic pressure (LVSP) estimation and corrected MW analysis methods. Transthoracic echocardiography was performed in all patients at baseline and 3–6 months after SM. Preoperative and postoperative parameters such as global work index (GWI), global constructive work (GCW), global wasted work (GWW), and global work efficiency (GWE) were analyzed to investigate the characteristics of LV myocardial mechanical remodeling. The degree of histological MF was evaluated to determine the correlation between corrected MW parameters and MF.

**Results:**

Noninvasive LVSP estimated by adding systolic blood pressure to the peak LV outflow tract gradient was well consistent with invasively measured LVSP (*r* = 0.98, *P* < 0.001; ICC = 0.96, *P* < 0.001). After SM, GWI, GCW, and GWE were significantly decreased (all *P* < 0.001), and GWW was significantly increased in HOCM patients (*P* = 0.002). Postoperatively, all patients exhibited new-onset complete left bundle branch block. Corrected GWI (R²=0.22, *P* = 0.002) and GCW (R²=0.25, *P* < 0.001) were independently associated with the extent of MF.

**Conclusion:**

We validated a corrected method for analyzing MW in HOCM patients. HOCM patients may experience reduced metabolism and compromised contraction coordination after SM. GWI and GCW are associated with the level of MF.

**Supplementary Information:**

The online version contains supplementary material available at 10.1186/s44156-026-00113-7.

## Graphical Abstracts

**Supplementary Information:**

The online version contains supplementary material available at 10.1186/s44156-026-00113-7.

## Graphical abstract

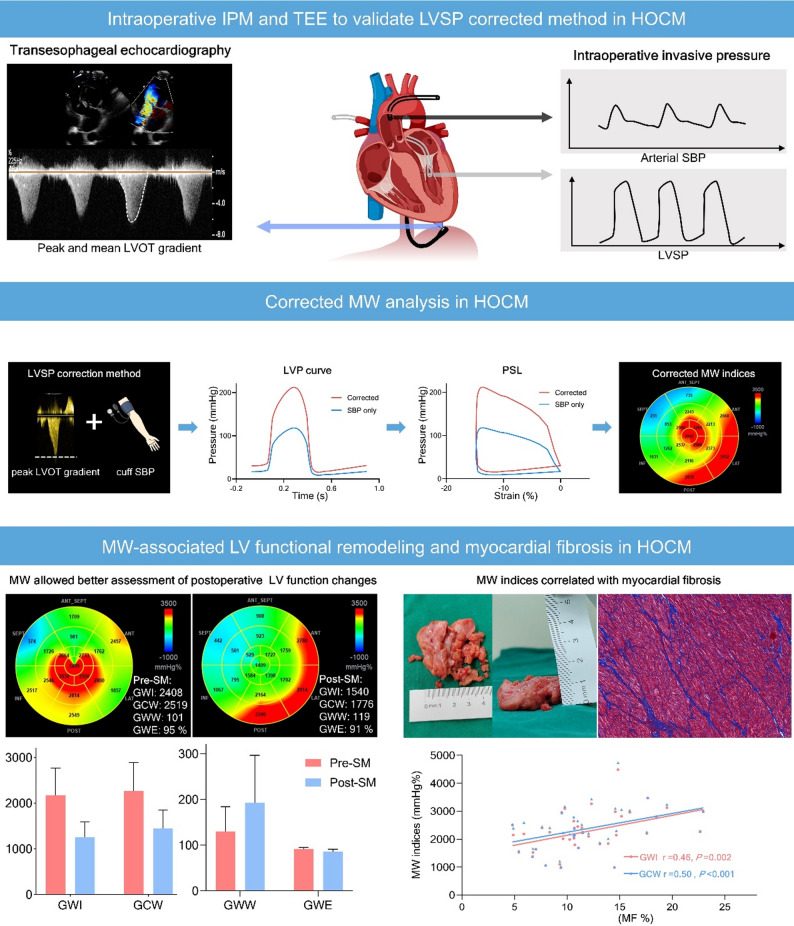

## Introduction

Hypertrophic obstructive cardiomyopathy (HOCM), which is reported in a significant proportion (approximately 60–70% in some classical cohorts) of hypertrophic cardiomyopathy (HCM) patients, is characterized by left ventricular outflow tract (LVOT) obstruction and increased afterload [1–3]. This state of chronic pressure overload could lead to left ventricular (LV) dysfunction in the HOCM patients [4]. Echocardiography is the mainstream imaging modality for assessing LV function in HOCM patients [5, 6]. Nevertheless, echocardiographic parameters as LV ejection fraction (LVEF) and global longitudinal strain (GLS) may be influenced by load dependence [7, 8].

Myocardial work (MW) is a novel echocardiographic technique that allows assessing LV function by incorporating afterload [9, 8]. However, there are limitations in applying traditional MW analysis methods in HOCM as LVSP does not equal systolic blood pressure (SBP) owing to the LVOT obstruction [8, 10]. As increased afterload is their main haemodynamic characteristic and plays a pivotal role in the physiological pathologies of HOCM, we assume that MW, if properly corrected, may serve as an important tool for the assessment of cardiac function and MF in HOCM. It has been shown that corrected MW can be utilised to assess LV function in aortic stenosis with similarly increased afterload [11, 12]. Multiple studies have found that MW parameters are valuable in non-obstructive HCM, particularly in assessing MF [13–15]. Moreover, it has been confirmed that MW indices in other various cardiac conditions, such as patients with dilated cardiomyopathy, hypertension, heart failure, and arrhythmia are advantageous [16–18, 12]. This study aimed to (1) validate a non-invasive correction method for MW calculation, (2) to delineate the cardiac functional alterations following septal myectomy (SM) utilizing the corrected MW indices, and (3) to explore the association of MW indices with MF.

## Methods

### Patient population

**Fig. 1:**
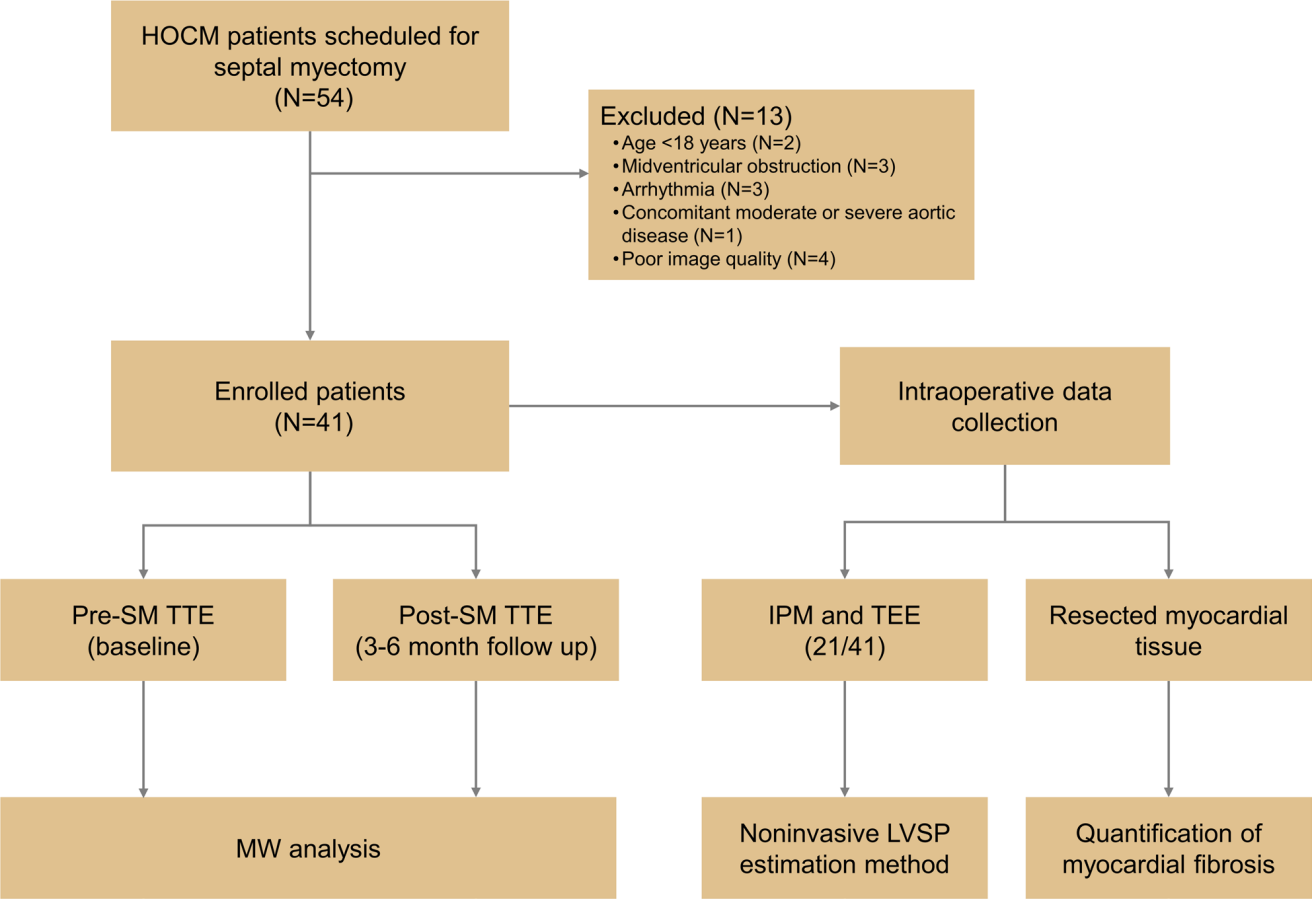
Study flow chart. Enrollment and disposition of patients. HOCM= hypertrophic obstructive cardiomyopathy; IPM=invasive pressure measurement; LVSP= left ventricular systolic pressure; SM= septal myectomy; TEE= transesophageal echocardiography

This study prospectively enrolled 54 consecutive HOCM patients who were scheduled for SM from September 2021 to April 2023. HOCM was diagnosed according to the up-to-date guidelines [3, 19, 5]. All patients met the criteria for SM with unsatisfactory symptoms despite optimal medical treatment and LVOT gradients of ≥ 50 mmHg at rest or after provocation (Valsalva maneuver or exercise). The exclusion criteria included age < 18 years, midventricular obstruction (defined as midventricular gradient ≥ 30 mmHg at rest), arrhythmia, concomitant moderate or severe aortic disease, and poor image quality (defined as the inability to obtain myocardial strain for more than one segment even after manual adjustment). HOCM patients presenting with midventricular obstruction were not included, due to the significant variability in the LV intracavitary pressure. 41 HOCM patients were ultimately enrolled in this research (Fig. 1).

All patients received a comprehensive transthoracic echocardiography (TTE) within 1 month before, and 3–6 months after the SM. The inpatient electronic medical record system was used to collect demographic and clinical data. In addition, invasive pressure measurements (IPM) and transesophageal echocardiography (TEE) were carried out during the SM procedure. The excised myocardial tissue was routinely obtained for pathologic examination after surgery. The study protocol adhered to the tenets of the Declaration of Helsinki and the ethical committee of Tongji Medical College, Huazhong University of Science and Technology approved the protocol. Informed consent was obtained from all patients.

### Intraoperative invasive pressure measurement and transesophageal echocardiography

To validate the feasibility of using LVOT gradient to correct arterial SBP for the analysis of corrected MW indices, the LVSP and arterial SBP were invasively measured in the operating room, similar to the approaches reported by Schizas et al. [20].

**Fig. 2:**
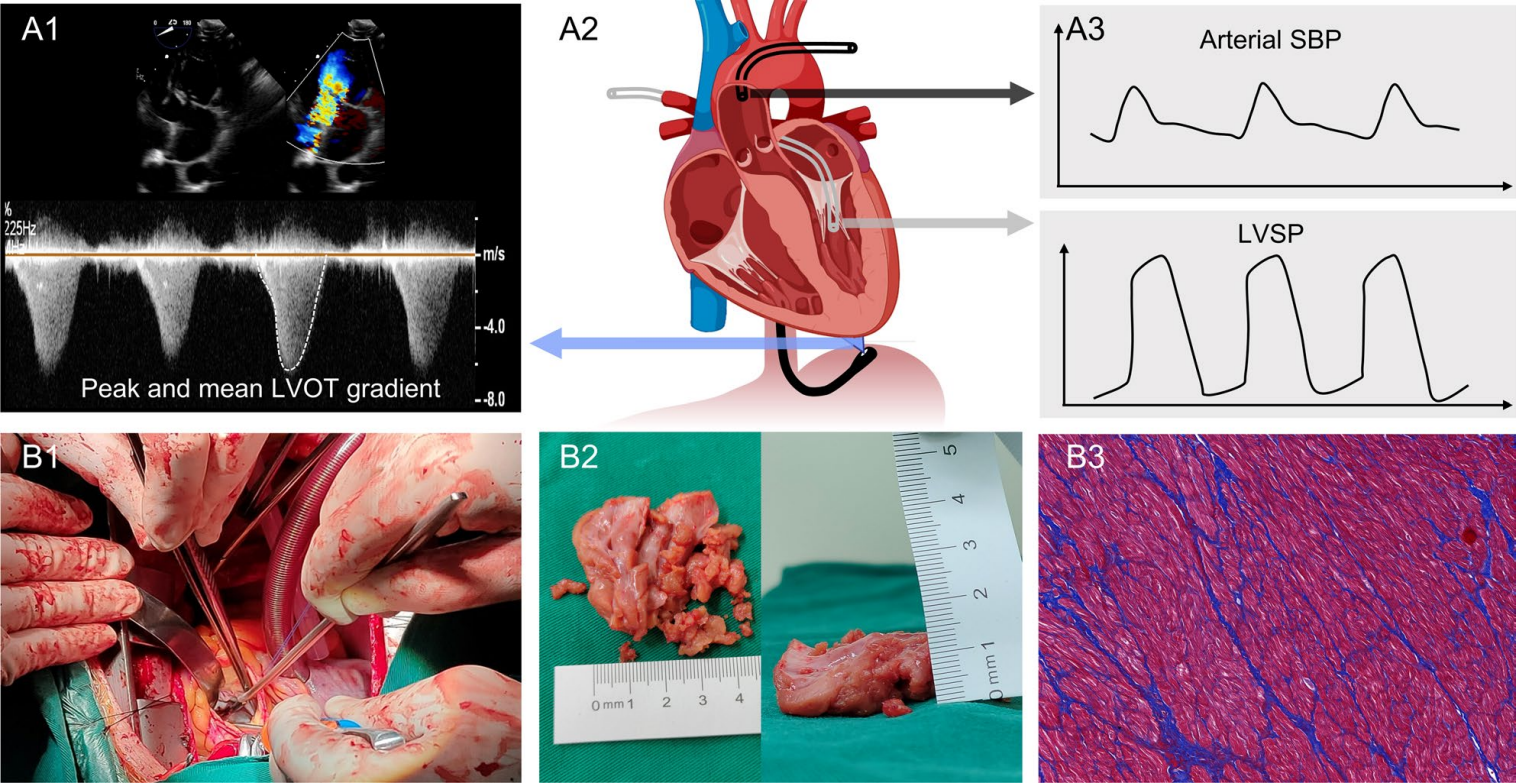
Schematic of intraoperative invasive pressure and histological MF measurement. (**A1-A3**) Two catheters was inserted into the left ventricle (gray) and the ascending aorta (black) for invasive pressure measurements. In parallel, continuous wave Doppler spectrum of the LVOT obtained by transesophageal echocardiography and the peak and mean gradients was measured. (**B1-B3**) Myocardial tissues were collected after septal myectomy procedure and the degree of MF was analyzed against pathological staining graphs after Masson’s trichrome stain. LVOT= left ventricular outflow tract; MF=myocardial fibrosis; SBP= systolic blood pressure; Other abbreviations as in Fig. 1

Intraoperative IPM was performed as follows: after median sternotomy under anesthesia, cardiopulmonary bypass (CPB) was established by cannulating the right atrium and ascending aorta. One pressure catheter was placed via right superior pulmonary vein to the LV to measure LVSP (LVSP_cath_), and another pressure catheter was connected to the antegrade cardioplegia catheter inserted in the ascending aorta to measure arterial SBP before the institution of CPB. The other end of the two fluid-filled pressure catheters connected to an external pressure transducer (Meritrrans, DTXPlusTM DT-4812), which was calibrated with the midaxillary line as the zero level. Catheter pressure was displayed in real time by a cardiac monitor (Mindray, BeneVision N15) connected to the pressure transducer. LVSP and arterial SBP were recorded after the pressure curve stabilized. During the process of intraoperative IPM, we also acquired LVOT flow spectrum in the deep transgastric 5-chamber view using continuous wave Doppler using TEE. Peak and mean gradients of LVOT were measured after the procedure by an experienced echocardiographer through tracing the spectrum border (Fig. 2A1-A3).

To explore a noninvasive approach for evaluating LVSP, we computed the estimated LVSP through three distinct methods: by summing the peak LVOT gradient with arterial SBP (LVSP_peak_), by summing the mean LVOT gradient with arterial SBP (LVSP_mean_), and by summing the average of peak and mean LVOT gradients with arterial SBP (LVSP_arg_). The LVSP_cath_ was set as the gold standard for comparing the feasibility and precision of LVSP_peak_, LVSP_mean_, and LVSP_arg_.

### Transthoracic echocardiography and electrocardiography

The standard images and measurements were completed based on current guidelines [21]. TTE was performed with the Vivid E95 ultrasound system. (GE Vingmed Ultrasound, Horten, Norway). Echocardiographic images were collected with an M5Sc probe triggered by an electrocardiogram, stored digitally in cine loop format and analyzed offline with a proprietary software (EchoPAC 204; GE Vingmed Ultrasound). Peak LVOT gradient at rest was assessed using continuous-wave Doppler from apical five-chamber view. Standard 12‑lead electrocardiography was performed in all patients preoperatively and postoperatively. Resting ECGs were recorded at a paper speed of 25 mm/s and calibration of 10 mm/mV, with patients in the supine position.

### Myocardial work analysis

MW analysis was performed using proprietary software (EchoPAC version 204, GE Vingmed Ultrasound). The brachial blood pressure of each patient was measured 3 times during acquisition of apical views with an electronic sphygmomanometer (Panasonic, EW3106), and the average value was used for offline MW analysis. For preoperative HOCM patients, noninvasive LVSP substitute (the sum of resting peak LVOT gradient and cuff SBP, confirmed by the IPM data as the most accurate noninvasive surrogate for LVSP) was entered into the software to calculate the corrected MW indices. The additional steps of the MW analysis in preoperative HOCM patients followed the traditional method [8, 9] (online supplemental material Fig. 1). We also analyzed uncorrected MW parameters (derived using peripheral SBP only) to compare with the corrected MW parameters (derived using estimated LVSP, which was obtained by adjusting peripheral SBP with the peak LVOT gradient). Postoperative MW analysis was performed using conventional method (peripheral SBP only) because LVOT obstruction was released after SM. MW analysis allowed the noninvasive construction of the LV pressure-strain loop and produced MW indices: global work index (GWI), global constructive work (GCW), global wasted work (GWW), and global work efficiency (GWE).

GWI represents the total work performed by the LV throughout the cardiac cycle (systole and isovolumic relaxation), calculated as the total area within the LV pressure-strain loop. It provides an overall measure of LV energy expenditure. GCW quantifies the work contributing positively to LV ejection. It includes work performed during myocardial shortening in systole (positive strain) and work performed during lengthening in isovolumic relaxation (negative strain). GCW is a component of GWI that reflects effective work. GWW measures the work that does not contribute to effective ejection, representing an energy loss. It comprises work performed during lengthening in systole (negative strain) and during shortening in isovolumic relaxation (positive strain). Minimizing GWW is associated with higher efficiency. GWE is expressed as a percentage and represents the ratio of constructive work to the total work performed. It is calculated as GWE = [GCW / (GCW + GWW)] * 100%. GWE directly reflects the efficiency of the LV in converting myocardial work into effective ejection [8, 9].

### Myocardial histopathology

Myocardial tissue was obtained from each patient after septal myectomy. Tissue samples were promptly fixed in 10% buffered formalin, subsequently embedded in paraffin, sectioned at a thickness of 4 μm along the longitudinal myocardial fascicles, and stained using the Masson trichrome method to evaluate the extent of MF (Fig. 2B1-B3). To assess MF, regions of fibrosis located in subendocardial or perivascular areas were excluded. Subsequently, 4 to 5 fields were selected from digital slide images at 200× magnification, obtained using the Pannoramic MIDI scanner (3D HISTECH, Hungary). The degree of MF (the ratio of the MF area to the cross-sectional area) was quantified using Image J software (Image J 1.53, NIH, USA). The image processing methodology concentrated on establishing color thresholds, differentiating collagen from myocardial tissue, and implementing filters to remove artifacts. The mean value of the four to five fields was computed as the mean MF for each sample. All tissue samples were evaluated by an observer who was blinded to the echocardiographic results.

### Statistical analysis

Continuous variables were summarized using the mean ± standard deviation (SD) or the median along with the interquartile range (IQR). Categorical values were presented as counts and percentages. The correlation between LVSPcath and estimated LVSP (including LVSP_peak_, LVSP_mean_, and LVSP_arg_), as well as the correlation between MW parameters and the degree of MF, were analyzed using Pearson’s or Spearman’s correlation tests, contingent upon the data distribution. The agreements of estimated LVSP and LVSP_cath_ were evaluated using intraclass correlation coefficients (ICC) and Bland-Altman analysis. Differences in echocardiographic, electrocardiographic, and MW indices before and after the surgery were assessed using the paired t test, the Wilcoxon signed rank test, or the χ2 test, as appropriate. The relationship between the associated factors and histological MF was assessed using univariate linear regression. Significant variables from univariate analyses were included in multivariable analyses. Collinearity was assessed using the variance inflation factor, with values below 5 indicating a low likelihood of collinearity. 20 HOCM patients were chosen at random to evaluate intra- and interobserver agreement of MW analysis using ICC and Bland-Altman analysis. The second observer was blinded to the first observer’s measurements, and the first observer repeated the analysis after 1 month. A two-tailed *P* value less than 0.05 was deemed to indicate statistical significance. Statistical analyses were conducted utilizing R software, version 4.3.1 (R Foundation).

## Results

### Patients

Among the 41 patients in this study, the age at the time of operation was 53(40–60) years, and 27 (65.9%) patients were men. 30 (73.2%) patients had New York Heart Association class III/IV symptoms. Arterial hypertension was present in 11 (26.8%) patients, and diabetes mellitus in 2 (4.9%). Other clinical characteristics are summarized in Table 1. All patients underwent successful SM and were discharged uneventfully.

**Table 1:** Baseline clinical characteristics

	All patients (*n* = 41)
Age, y	53(40–60)
Male, n (%)	27(65.9)
Body surface area, m²	1.8 ± 0.2
Body mass index, kg/m²	24.5(23.3–26.6)
Systolic blood pressure, mmHg	124 ± 15
Diastolic blood pressure, mmHg	79 ± 11
Heart rate, bpm	69 ± 10
Maximal LVH, mm	23.7 ± 3.0
MF, %	13.7 ± 2.7
NYHA functional class, n (%)	
II	11(26.8)
III/IV	30(73.2)
Family history of HCM, n (%)	8(19.5)
Arterial hypertension, n (%)	11(26.8)
Diabetes mellitus, n (%)	2(4.9)
Smoking history, n (%)	19(46.3)
Alcohol history, n (%)	19(46.3)
Syncope history, n (%)	6(14.6)
Creatinine, µmol/L	71.0 ± 18.1
Ureanitrogen, mmol/L	6.6 ± 1.8
Albumin, g/L	40.0 ± 3.6
Globulin, g/L	22.6 ± 3.3
Glomerular filtration rate, ml/(min/1.73 m²)	100.6(87.4-109.4)
ALT, U/L	23(17–31)
AST, U/L	26.0(23–37)
Medications, n (%)	
β blockers	30(73.2)
Calcium channel blockers	24(58.5)
ICD implantation, n (%)	2(4.9)

Values are mean ± SD, n (%), or median (IQR)

ALT= Alanine aminotransferase; AST= aspartate aminotransferase; HCM= hypertrophic cardiomyopathy; ICD= Implantable cardioverter-defibrillator; LVH= left ventricular hypertrophy; MF=myocardial fibrosis; NYHA = New York Heart Association

### Conventional echocardiography and electrocardiographic changes

**Fig. 3:**
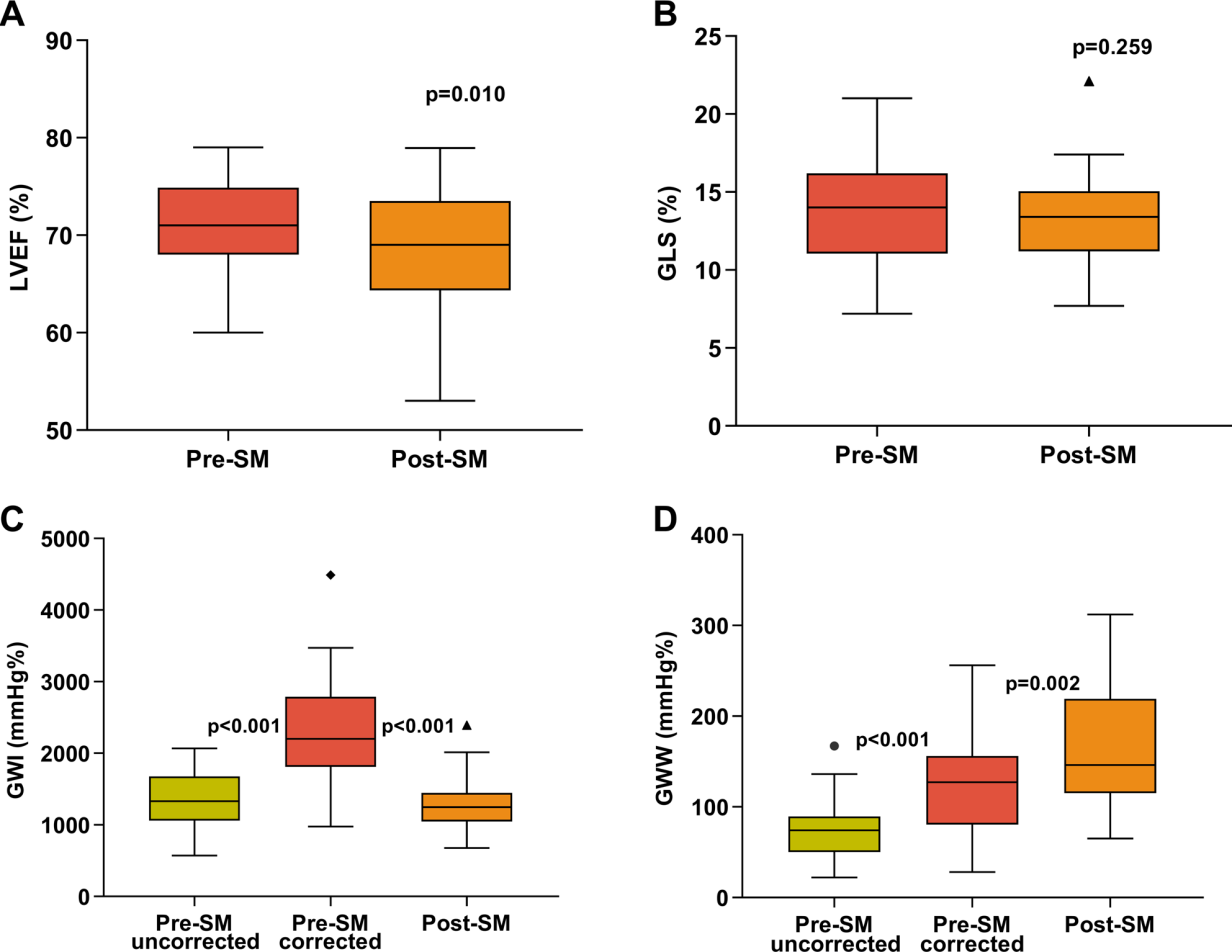
Box plots with Tukey whiskers of LV functional parameters. Comparisons of pre- and postoperative LVEF (**A**), GLS (**B**), GWI (**C**), and GWW (**D**). GLS= global longitudinal strain; GWI= global work index; GWW= global wasted work; LVEF= left ventricular ejection fraction; Other abbreviations as in Figs. 1

Table 2 provides the details of echocardiographic parameters before and 3–6 months after SM. Following the operation, the interventricular septal thickness was significantly reduced from 21.2 ± 2.9 mm to 15.0 ± 2.5 mm (*P* < 0.001). The grade of mitral regurgitation and incidence of mitral valve systolic anterior motion significantly decreased (*P* < 0.001). The preoperative resting peak LVOT gradient was 84.8 ± 35.3 mmHg, which significantly decreased to 11.4 ± 5.2 mmHg after SM (*P* < 0.001). There was a mild but significant reduction in LVEF (*P* = 0.010), while no significant change in LV GLS (*P* = 0.259) after SM (Fig. 3A and B). Electrocardiographic findings revealed that all patients developed new-onset complete left bundle branch block (LBBB) following surgery. Postoperative QRS duration was significantly prolonged compared with the preoperative baseline [106 (101–114) vs. 161 (154–171), *P* < 0.001].

**Table 2:** Pre- and post-operative echocardiographic characteristics in 41 HOCM patients

	Pre-SM	Post-SM	*P* value
LAD, mm	44.5 ± 5.9	41.5 ± 5.8	< 0.001
LAV, ml	77.5 ± 27.9	56.7 ± 19.2	< 0.001
LAEF^a^, %	47.8 ± 11.2	50.5 ± 12.7	0.191
IVST, mm	21.2 ± 2.9	15.0 ± 2.5	< 0.001
LVEDV, ml	82.8 ± 20.8	77.3 ± 20.5	0.033
LVESV, ml	24.0 ± 7.0	24.5 ± 8.7	0.954
SV, ml	58.3 ± 16.0	52.8 ± 14.2	0.005
LVEF^a^, %	71.0 ± 4.6	66.7 ± 6.0	0.010
E, m/s	0.8(0.6–0.9)	0.7(0.5–1.1)	0.657
A, m/s	0.8(0.6–1.1)	0.9(0.7–1.3)	0.036
Mitral E/A	1.0(0.7–1.3)	0.8(0.6–0.9)	0.001
e septum, cm/s	5.0(3.5-6.0)	4.0(3.0–5.0)	< 0.001
e lateral, cm/s	6.0(5.0–8.0)	7.0(6.0–9.0)	0.029
E/e	14.3(12.0-18.4)	12.5(10.0–20.0)	0.275
MR ≥ 2	35(85.4)	1(2.4)	< 0.001
SAM, n (%)	36(87.8)	4(9.8)	< 0.001
Resting peak LVOT gradient, mm Hg	84.8 ± 35.3	11.4 ± 5.2	< 0.001
LV GLS, %	13.9 ± 3.8	13.4 ± 2.8	0.259

Values are mean ± SD, n (%), or median (IQR). ^a^ LAEF and LVEF were measured with biplane method

IVST= Interventricular septum thickness; LAD= Left atrium diameter; LAEF= Left atrium ejection fraction; LAV= Left atrium volume; LV GLS= Left ventricular global longitudinal strain; LVEDV= Left ventricular end-diastolic volume; LVEF= Left ventricular ejection fraction; LVESV= Left ventricular end-systolic volume; LVOT= Left ventricular outflow tract; MR= Mitral regurgitation (MR ≥ 2 indicates moderate or greater MR); SAM= Systolic anterior motion; SM= septal myectomy; SV= Stroke volume

### IPM data analysis

We successfully performed IPM in 21 of 41 HOCM patients. IPM was precluded in 20 patients due to unsuitability of insertion of an antegrade cardioplegia catheter for perfusion of cardioplegic solution (*n* = 7) or suboptimal LVOT flow spectrum collection from TEE despite adjustment by the echocardiographer(*n* = 13). LVSP_cath_ was recorded as 156 ± 44 mmHg. LVSPpeak, LVSPmean and LVSParg were observed to be 163 ± 50 mmHg, 122 ± 26 mmHg, and 142 ± 37 mmHg, respectively. The results demonstrate that LVSP_peak_ is the most accurate noninvasive substitute (*r* = 0.98, *P* < 0.001; ICC = 0.96, *P* < 0.001; mean difference=-7.1 mmHg; 95% limits of agreement= -29.0 to 14.8 mmHg) that approximates the LVSP_cath_ (online supplemental material Fig. 2). Therefore, we employed this methodology, incorporating the resting peak LVOT gradient and cuff SBP as a noninvasive proxy for LVSP, to assess corrected MW parameters in HOCM (online supplemental material Fig. 3).

### Myocardial work indices

The comparison of MW indices is summarized in Table 3. Compared to the pre-SM uncorrected MW indices, the pre-SM corrected MW indices showed significant increases in GWI (Fig. 3C), GCW, and GWW (all *P* < 0.001), while GWE remained unchanged.

**Table 3:** Comparison of pre- and post-operative myocardial work parameters in 41 HOCM patients

	Pre-SM		Post-SM
	uncorrected	corrected	
GWI (mmHg%)	1339 ± 404	2263 ± 599^**†**^	1273 ± 349^**‡**^
GCW (mmHg%)	1368(1121–1738)	2299(1947–2880)^**†**^	1389(1175–1744)^**‡**^
GWW (mmHg%)	74(50–90)	127(81–156)^**†**^	146(115–219)^**‡**^
GWE (%)	92(90–95)	92(90–95)	89(84–91)^**‡**^

Values are mean ± SD or median (IQR). ^**†**^*P* < 0.05 (Pre-SM corrected MW indices vs. Pre-SM uncorrected MW indices); ^**‡**^*P* < 0.05 (Pre-MW corrected MW indices vs. Post-SM MW indices)

GCW= global constructive work; GWE= global work efficiency; GWI= global work index; GWW= global wasted work; MW= myocardial work; SM= septal myectomy

In comparison to pre-SM corrected MW indices, post-SM MW indices had a significant reduction in GWI and GCW (all *P* < 0.001). Moreover, postoperative GWW (Fig. 3D) exhibited a further increase (*P* = 0.002), while GWE decreased concurrently (*P* < 0.001).

### Associations between histological MF and corrected MW indices

**Fig. 4:**
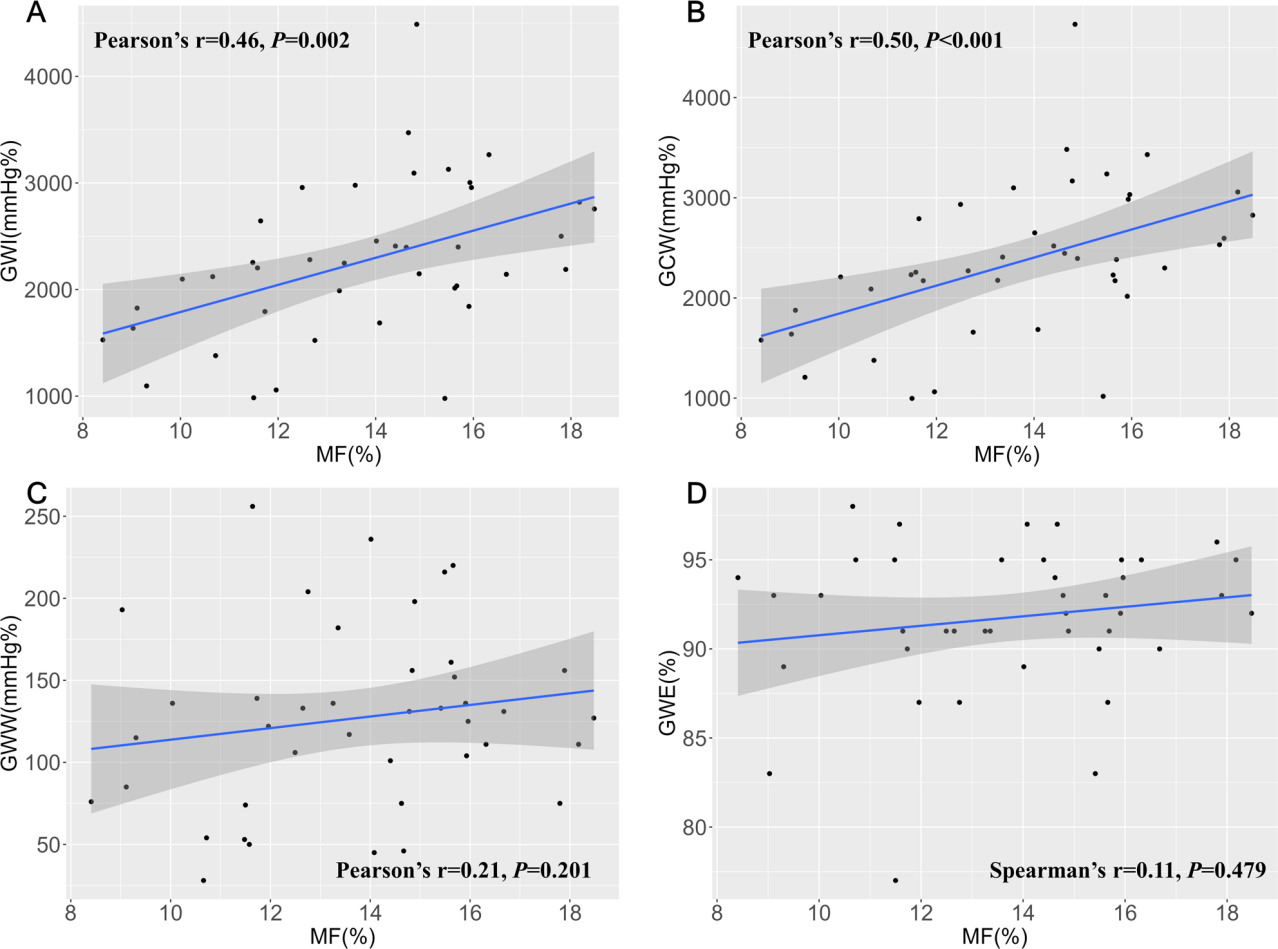
The correlation between the MW indices and myocardial fibrosis. Only GWI (**A**) and GCW (**B**) were significantly correlated with myocardial fibrosis. GCW= global constructive work; GWE= global work efficiency. Other abbreviations as in Figs. 2 and 3

The mean degree of histological MF in 41 patients was 13.7 ± 2.7%. Pre-SM corrected GWI (*r* = 0.46, *P* = 0.002) and GCW (*r* = 0.50, *P* < 0.001) were significantly correlated with the degree of histological MF, while GWW (*r* = 0.20, *P* = 0.201) and GWE (*r* = 0.11, *P* = 0.479) were not (Fig. 4).

Univariate linear regression analysis indicated that maximal LVH (R²=0.15, *P* = 0.012), resting peak LVOT gradient (R²= 0.21, *P* = 0.002), GWI (R²=0.22, *P* = 0.002), and GCW (R²=0.25, *P* < 0.001) are associated with the degree of histological MF. To prevent collinearity issues, we conducted separate multivariate linear regression analyses for GWI and GCW. The multivariate analysis found that GWI (*P* = 0.038) and GCW (*P* = 0.021) were independently associated with the level of MF (Table 4).

**Table 4:** Regression analysis on histological MF in patients with HOCM

	Univariate		Multivariate (with GWI)		Multivariate (with GCW)	
	β(95%CI)	*P*	β(95%CI)	*P*	β(95%CI)	*P*
Age, y	-0.02 (-0.09 to 0.06)	0.620				
Male, n (%)	-0.15 (-1.94 to 1.64)	0.869				
Body mass index, kg/m²	0.19 (-0.07 to 0.46)	0.159				
Smoking history, n (%)	0.50 (-1.20 to 2.20)	0.555				
Alcohol history, n (%)	-1.08(-2.75 to 0.59)	0.199				
Maximal LVH, mm	0.34 (0.08 to 0.60)	0.012	0.13 (-0.18 to 0.44)	0.395	0.13 (-0.17 to 0.44)	0.384
LAD, mm	0.05 (-0.10 to 0.19)	0.497				
LVEF, %	-0.07 (-0.25 to 0.12)	0.462				
Resting peak LVOT gradient, mmHg	0.03 (0.01 to 0.06)	0.002	0.02 (-0.01 to 0.05)	0.216	0.02 (-0.01 to 0.04)	0.257
LV GLS, %	0.11 (-0.12 to 0.33)	0.336				
GWI (mmHg%)	0.002 (0.001 to 0.003)	0.002	0.001 (0.0001 to 0.0023)	0.038		
GCW (mmHg%)	0.002 (0.001 to 0.003)	< 0.001			0.001 (0.0002 to 0.0023)	0.021
GWW (mmHg%)	0.008 (-0.01 to 0.02)	0.289				
GWE (%)	0.11 (-0.10 to 0.31)	0.292				

Abbreviations as in Tables 1, 2 and 3

### Reproducibility

The MW indices demonstrated excellent reproducibility, which was proven by the high ICC (online supplemental material Table 1). Bland-Altman analysis showed good intra- and interobserver agreement and displayed as Bland-Altman Plots (online supplemental material Fig. 4).

## Discussion

In this study, we analyzed changes in LV function after SM and the relationship between corrected MW parameters and MF in HOCM patients. The key findings are summarized as follows: (1) Corrected MW can be used to evaluate LV function in HOCM through noninvasive estimation of LVSP, achieved by summing the LVOT gradient and SBP. (2) Following SM, HOCM patients experienced a significant decrease in GWI, GCW, and GWE and a significant increase in GWW, which demonstrates a reduction in myocardial workload and compromised coordination of myocardial contraction; (3) Corrected GWI and GCW independently associated with the extent of histological MF in HOCM patients.

### Corrected MW indices in HOCM

To make MW available for cardiac function assessment among patients with HOCM, our study started by addressing the challenges of non-invasively measuring LVSP. Our results revealed that non-invasive LVSP among patients with HOCM could be accurately estimated by adding the resting peak LVOT gradient to arterial SBP, enabling subsequently MW analysis. Nevertheless, this method differs from a recent study, which used the average of the peak and mean LVOT gradient to correct SBP [22]. This discrepancy may be attributed to differences in pressure measurement schemes. Considering our results have shown that LVSP_peak_ was slightly better than LVSP_arg_ and obtaining the peak LVOT gradient was more straightforward, the LVSP_peak_ was finally preferred in this study. In this investigation, the corrected myocardial work indices, including GWI, GCW, and GWW, exhibited higher values compared to those obtained through the uncorrected method. This suggests that the LV has to work harder to pump blood into ascending aorta due to the narrowing of the LVOT. The increased workload can lead to higher energy demands on the heart [23, 24], which finally lead to symptoms of cardiac insufficiency.

### MW evaluation of postoperative LV myocardial mechanical remodeling

Prior studies demonstrated that MW parameters provide valuable insights into LV systolic performance and efficiency [8, 7]. However, to our understanding, there have been no studies that have utilized MW to evaluate LV functional alterations following SM among patients with HOCM. In this study, we found that GWI and GCW were significantly reduced after SM, which indicate a significant reduction in LV workload. These findings are consistent with prior literature demonstrating that myocardial metabolism is enhanced in HOCM patients due to increased afterload and is significantly reduced after LVOT obstruction relief [24]. Intriguingly, we noted a further increase in GWW and a subsequent decrease in GWE in postoperative HOCM patients, indicating compromised LV wall motion coordination after SM. This could be attributed to a usual complication of SM, LBBB [3, 25]. Complete LBBB developed in all HOCM patients after SM. This conduction disturbance may alter regional LV mechanics, as previous studies have demonstrated that LBBB disrupts the coordinated contraction of the septal and lateral walls, resulting in increased wasted work and diminished myocardial efficiency [26, 8]. These findings demonstrated the superiority of MW parameters in evaluating myocardial mechanical remodeling in HOCM patients following SM. In contrast, mild decreased LVEF and unchanged LV GLS, as load-dependent measures, seems to be inadequate in reflecting clinical symptom improvement and had restricted utility in assessing LV remodeling.

### Corrected MW indicator associated with MF

Recently, several studies have investigated the value of MW indices in the assessment of MF in nonobstructive HCM [27, 14, 13]. The results showed a negative correlation between MW indices and the degree of MF [27, 14]. However, the value of MW indices in the assessment of MF among patients with HOCM has not been extensively investigated. Our results showed that corrected GWI and GCW were independently associated with the level of MF in HOCM patients. This could be attributed to the patients with HOCM having significantly increased afterload due to LVOT obstruction. Increased GWI and GCW in HOCM patients are primarily caused by elevated afterload, which has been linked to increased MF severity in previous research [28, 29]. We explored using MW indices to assess MF in HOCM patients and found them to be useful.

### Clinical implications

Including MW indices in the standard evaluation of patients with HOCM can improve our understanding of their cardiac function, as increased afterload is their main hemodynamic characteristic. This study investigated the effectiveness of corrected MW indices in evaluating postoperative myocardial mechanical remodeling and the level of MF in HOCM patients. Our study demonstrated that MW parameters can effectively evaluate postoperative cardiac function changes as opposed to LVEF and LV GLS. Postoperative alterations in MW parameters showed that cardiac workload in HOCM patients is significantly mitigated after SM procedure. Additionally, a potential impairment in LV movement coordination may occur after SM, which may contribute to the lack of expected improvement in postoperative symptoms among certain individuals with HOCM. Furthermore, MW parameters could serve as a valuable adjunct for non-invasive evaluation of MF severity. Based on these preliminary findings, future research could further explore the potential uses of MW in HOCM, including the stratification of sudden death risk, surgical decision making and prognosis prediction. MW may improve the understanding and management of HOCM, potentially leading to better patient outcomes as diagnosis and treatment evolve [30].

### Study limitations

First, this study is limited to one center and a small sample size and the invasive pressure assessments were not possible in 50% of the patients, which may lead to selection bias and may limit the generalizability of our findings. Second, utilizing the peak LVOT gradient and arterial SBP for non-invasive estimation of LVSP is suboptimal due to temporal phase misalignment. Nevertheless, our research has demonstrated that a combination of these measurements can yield a satisfactory approximation of LVSP. Third, during anesthesia for patients with HOCM, pharmacological agents can affect cardiac function and hemodynamic status, potentially deviating from their awake state. However, using IPM data to validate a noninvasive method for estimating LVSP is acceptable. Larger scale, multi-center studies are needed to validate our findings and to explore the clinical implications of MW indices in the broader HOCM patient population.

## Conclusions

We validate a corrected method for echocardiographic MW analysis in HOCM and demonstrate that MW indices could improve our understanding of LV function changes after SM in HOCM patients. HOCM patients undergoing SM have decreased myocardial metabolism and compromised myocardial contraction coordination. Additionally, MW has potential value in the assessment of MF among patients with HOCM. Understanding MW in HOCM may contribute to a better assessment of the LV functional status and guide the appropriate management strategies for individuals affected by this condition.

## Supplementary Information

Below is the link to the electronic supplementary material.

Supplementary Material 1

## Data Availability

The datasets generated or analyzed during the study are available from the corresponding author on reasonable request.

## Abbreviations

CPB: Cardiopulmonary bypass
GCW: Global constructive work
GLS: Global longitudinal strain
GWE: Global work efficiency
GWI: Global work index
GWW: Global wasted work
HCM: Hypertrophic cardiomyopathy
HOCM: Hypertrophic obstructive cardiomyopathy
ICC: Intraclass correlation coefficients
IPM: Invasive pressure measurement
LV: Left ventricular
LVEF: Left ventricular ejection fraction
LVOT: Left ventricular outflow tract
LVP: Left ventricular pressure
LVSP: Left ventricular systolic pressure
MF: Myocardial fibrosis
MW: Myocardial work
PSL: Pressure strain loop
SBP: Systolic blood pressure
SM: Septal myectomy
TEE: Transesophageal echocardiography
TTE: Transthoracic echocardiography

## Declarations

### Generative AI and AI-assisted technologies in the writing process

No AI tools were used in the preparation of this work.

## References

[1] Ommen SR, Semsarian C. Hypertrophic cardiomyopathy: a practical approach to guideline directed management. Lancet. 2021;398:2102–8. 10.1016/S0140-6736(21)01205-8. 34600606 10.1016/S0140-6736(21)01205-8

[2] Ommen SR, Ho CY, Asif IM, Balaji S, Burke MA, Day SM, Dearani JA, Epps KC, Evanovich L, Ferrari VA, AHA/ACC/AMSSM/, HRS/PACES/SCMR guideline for the management of hypertrophic cardiomyopathy. a report of the American Heart Association/American College of Cardiology Joint committee on clinical practice guidelines, Circulation. 2024;149: e1239–311. 10.1161/cir.0000000000001250 10.1161/CIR.0000000000001250 38718139

[3] Ommen SR, Mital S, Burke MA, Day SM, Deswal A, Elliott P, Evanovich LL, Hung J, Joglar JA, Kantor P, et al. 2020 AHA/ACC Guideline for the Diagnosis and Treatment of Patients With Hypertrophic Cardiomyopathy: A Report of the American College of Cardiology/American Heart Association Joint Committee on Clinical Practice Guidelines. J Am Coll Cardiol. 2020;76:e159–240. 10.1016/j.jacc.2020.08.045. 33229116 10.1016/j.jacc.2020.08.045

[4] Maron MS, Olivotto I, Zenovich AG, Link MS, Pandian NG, Kuvin JT, Nistri S, Cecchi F, Udelson JE, Maron BJ. Hypertrophic cardiomyopathy is predominantly a disease of left ventricular outflow tract obstruction. Circulation. 2006;114:2232–9. 10.1161/CIRCULATIONAHA.106.644682. 17088454 10.1161/CIRCULATIONAHA.106.644682

[5] Arbelo E, Protonotarios A, Gimeno JR, Arbustini E, Barriales-Villa R, Basso C, Bezzina CR, Biagini E, Blom NA, de Boer RA, et al. 2023 ESC Guidelines for the management of cardiomyopathies. Eur Heart J. 2023;44:3503–626. 10.1093/eurheartj/ehad194. 37622657 10.1093/eurheartj/ehad194

[6] Ramchand J, Fava AM, Chetrit M, Desai MY. Advanced imaging for risk stratification of sudden death in hypertrophic cardiomyopathy. Heart. 2020;106:793–801. 10.1136/heartjnl-2019-315176. 31949025 10.1136/heartjnl-2019-315176

[7] Moya A, Buytaert D, Penicka M, Bartunek J, Vanderheyden M. State-of-the-Art: Noninvasive Assessment of Left Ventricular Function Through Myocardial Work. J Am Soc Echocardiogr. 2023;36:1027–42. 10.1016/j.echo.2023.07.002. 37437670 10.1016/j.echo.2023.07.002

[8] Russell K, Eriksen M, Aaberge L, Wilhelmsen N, Skulstad H, Remme EW, Haugaa KH, Opdahl A, Fjeld JG, Gjesdal O, et al. A novel clinical method for quantification of regional left ventricular pressure-strain loop area: a non-invasive index of myocardial work. Eur Heart J. 2012;33:724–33. 10.1093/eurheartj/ehs016. 22315346 10.1093/eurheartj/ehs016 PMC3303715

[9] Roemer S, Jaglan A, Santos D, Umland M, Jain R, Tajik AJ, Khandheria BK. The Utility of Myocardial Work in Clinical Practice. J Am Soc Echocardiogr. 2021;34:807–18. 10.1016/j.echo.2021.04.013. 33895250 10.1016/j.echo.2021.04.013

[10] Klues HG, Leuner C, Kuhn H. Left ventricular outflow tract obstruction in patients with hypertrophic cardiomyopathy: increase in gradient after exercise. J Am Coll Cardiol. 1992;19:527–33. 10.1016/s0735-1097(10)80266-9. 1302453 10.1016/s0735-1097(10)80266-9

[11] Jain R, Bajwa T, Roemer S, Huisheree H, Allaqaband SQ, Kroboth S, Perez Moreno AC, Tajik AJ, Khandheria BK. Myocardial work assessment in severe aortic stenosis undergoing transcatheter aortic valve replacement. Eur Heart J Cardiovasc Imaging. 2021;22:715–21. 10.1093/ehjci/jeaa257. 33106854 10.1093/ehjci/jeaa257

[12] Fortuni F, Butcher SC, van der Kley F, Lustosa RP, Karalis I, de Weger A, Priori SG, van der Bijl P, Bax JJ, Delgado V, et al. Left Ventricular Myocardial Work in Patients with Severe Aortic Stenosis. J Am Soc Echocardiogr. 2021;34:257–66. 10.1016/j.echo.2020.10.014. 33181281 10.1016/j.echo.2020.10.014

[13] Peters M, Jan MF, Ashraf M, Sanders H, Roemer S, Schweitzer M, Adefisoye J, Galazka P, Jain R, Jahangir A, et al. Myocardial Work in Apical Hypertrophic Cardiomyopathy. J Am Soc Echocardiogr. 2023;36:1043–54. 10.1016/j.echo.2023.06.013. 37406714 10.1016/j.echo.2023.06.013

[14] Goncalves AV, Rosa SA, Branco L, Galrinho A, Fiarresga A, Lopes LR, Thomas B, Baquero L, Carmo MM, Ferreira RC. Myocardial work is associated with significant left ventricular myocardial fibrosis in patients with hypertrophic cardiomyopathy. Int J Cardiovasc Imaging. 2021;37:2237–44. 10.1007/s10554-021-02186-3. 33598850 10.1007/s10554-021-02186-3

[15] Hiemstra YL, van der Bijl P, El Mahdiui M, Bax JJ, Delgado V, Marsan NA. Myocardial Work in Nonobstructive Hypertrophic Cardiomyopathy: Implications for Outcome. J Am Soc Echocardiogr. 2020;33:1201–8. 10.1016/j.echo.2020.05.010. 32680744 10.1016/j.echo.2020.05.010

[16] Calvillo-Arguelles O, Thampinathan B, Somerset E, Shalmon T, Amir E, Steve Fan CP, Moon S, Abdel-Qadir H, Thevakumaran Y, Day J, et al. Diagnostic and Prognostic Value of Myocardial Work Indices for Identification of Cancer Therapy-Related Cardiotoxicity. JACC Cardiovasc Imaging. 2022;15:1361–76. 10.1016/j.jcmg.2022.02.027. 35926895 10.1016/j.jcmg.2022.02.027

[17] Wang CL, Chan YH, Wu VC, Lee HF, Hsiao FC, Chu PH. Incremental prognostic value of global myocardial work over ejection fraction and global longitudinal strain in patients with heart failure and reduced ejection fraction. Eur Heart J Cardiovasc Imaging. 2021;22:348–56. 10.1093/ehjci/jeaa162. 32820318 10.1093/ehjci/jeaa162

[18] Prinzen FW, Lumens J. Investigating myocardial work as a CRT response predictor is not a waste of work. Eur Heart J. 2020;41:3824–6. 10.1093/eurheartj/ehaa677. 32944764 10.1093/eurheartj/ehaa677

[19] Maron BJ, Desai MY, Nishimura RA, Spirito P, Rakowski H, Towbin JA, Rowin EJ, Maron MS, Sherrid MV. Diagnosis and Evaluation of Hypertrophic Cardiomyopathy: JACC State-of-the-Art Review. J Am Coll Cardiol. 2022;79:372–89. 10.1016/j.jacc.2021.12.002. 35086660 10.1016/j.jacc.2021.12.002

[20] Schizas N, Georgia N, Samiotis I, Angouras DC, Argiriou M. Intraoperative accurate assessment of septal myectomy for hypertrophic obstructive cardiomyopathy (HOCM). J Card Surg. 2022;37:3322–4. 10.1111/jocs.16744. 35801496 10.1111/jocs.16744

[21] Mitchell C, Rahko PS, Blauwet LA, Canaday B, Finstuen JA, Foster MC, Horton K, Ogunyankin KO, Palma RA, Velazquez EJ. Guidelines for Performing a Comprehensive Transthoracic Echocardiographic Examination in Adults: Recommendations from the American Society of Echocardiography. J Am Soc Echocardiogr. 2019;32:1–64. 10.1016/j.echo.2018.06.004. 30282592 10.1016/j.echo.2018.06.004

[22] Batzner A, Hahn P, Morbach C, Störk S, Maack C, Verheyen N, Gerull B, Frantz S, Seggewiss H. Non-invasive estimation of left ventricular systolic peak pressure: a prerequisite to calculate myocardial work in hypertrophic obstructive cardiomyopathy. Eur Heart J - Cardiovasc Imaging. 2024;25:213–9. 10.1093/ehjci/jead236. 37722375 10.1093/ehjci/jead236 PMC10824478

[23] Niki K, Sugawara M, Asano R, Oka T, Kondoh Y, Tanino S, Iwade K, Magosaki N, Kasanuki H, Hosoda S. Disopyramide improves the balance between myocardial oxygen supply and demand in patients with hypertrophic obstructive cardiomyopathy. Heart Vessels. 1997;12:111–8. 10.1007/BF02767128. 9496461 10.1007/BF02767128

[24] Aoyama R, Takano H, Kobayashi Y, Kitamura M, Asai K, Amano Y, Kumita SI, Shimizu W. Evaluation of myocardial glucose metabolism in hypertrophic cardiomyopathy using 18F-fluorodeoxyglucose positron emission tomography. PLoS ONE. 2017;12:e0188479. 10.1371/journal.pone.0188479. 29176885 10.1371/journal.pone.0188479 PMC5703458

[25] Zheng R, Dong Y, Wu S, Su L, Zhao D, Chen X, Cai B, Fang X, Vijayaraman P, Huang W. Conduction system pacing following septal myectomy: Insights into site of conduction block. J Cardiovasc Electrophysiol. 2022;33:437–45. 10.1111/jce.15362. 35028984 10.1111/jce.15362

[26] Aalen JM, Remme EW, Larsen CK, Andersen OS, Krogh M, Duchenne J, Hopp E, Ross S, Beela AS, Kongsgaard E, et al. Mechanism of Abnormal Septal Motion in Left Bundle Branch Block: Role of Left Ventricular Wall Interactions and Myocardial Scar. JACC Cardiovasc Imaging. 2019;12:2402–13. 10.1016/j.jcmg.2018.11.030. 30772230 10.1016/j.jcmg.2018.11.030

[27] Galli E, Vitel E, Schnell F, Le Rolle V, Hubert A, Lederlin M, Donal E. Myocardial constructive work is impaired in hypertrophic cardiomyopathy and predicts left ventricular fibrosis. Echocardiography. 2019;36:74–82. 10.1111/echo.14210. 30488501 10.1111/echo.14210

[28] Avegliano G, Politi MT, Costabel JP, Kuschnir P, Trivi M, Ronderos R. Differences in the extent of fibrosis in obstructive and nonobstructive hypertrophic cardiomyopathy. J Cardiovasc Med (Hagerstown). 2019;20:389–96. 10.2459/JCM.0000000000000800. 30994509 10.2459/JCM.0000000000000800

[29] Ellims AH, Iles LM, Ling Lh, Chong B, Macciocca I, Slavin GS, Hare JL, Kaye DM, Marasco SF, McLean CA, et al. A comprehensive evaluation of myocardial fibrosis in hypertrophic cardiomyopathy with cardiac magnetic resonance imaging: linking genotype with fibrotic phenotype. Eur Heart J - Cardiovasc Imaging. 2014;15:1108–16. 10.1093/ehjci/jeu077. 24819852 10.1093/ehjci/jeu077

[30] Moody WE, Elliott PM. Changing concepts in heart muscle disease: the evolving understanding of hypertrophic cardiomyopathy. Heart. 2022;108:768–73. 10.1136/heartjnl-2021-320145. 35459726 10.1136/heartjnl-2021-320145

