# Supplementary material for: Evaluating left ventricular function and myocardial fibrosis in hypertrophic obstructive cardiomyopathy with corrected myocardial work
Source: Echo Res Pract. 2026 Apr 3;13:10. doi: 10.1186/s44156-026-00113-7 (PMC13047827; doi:10.1186/s44156-026-00113-7)
Supplement: Supplementary file 1 — Supplementary Material 1 [file 44156_2026_113_MOESM1_ESM.docx]

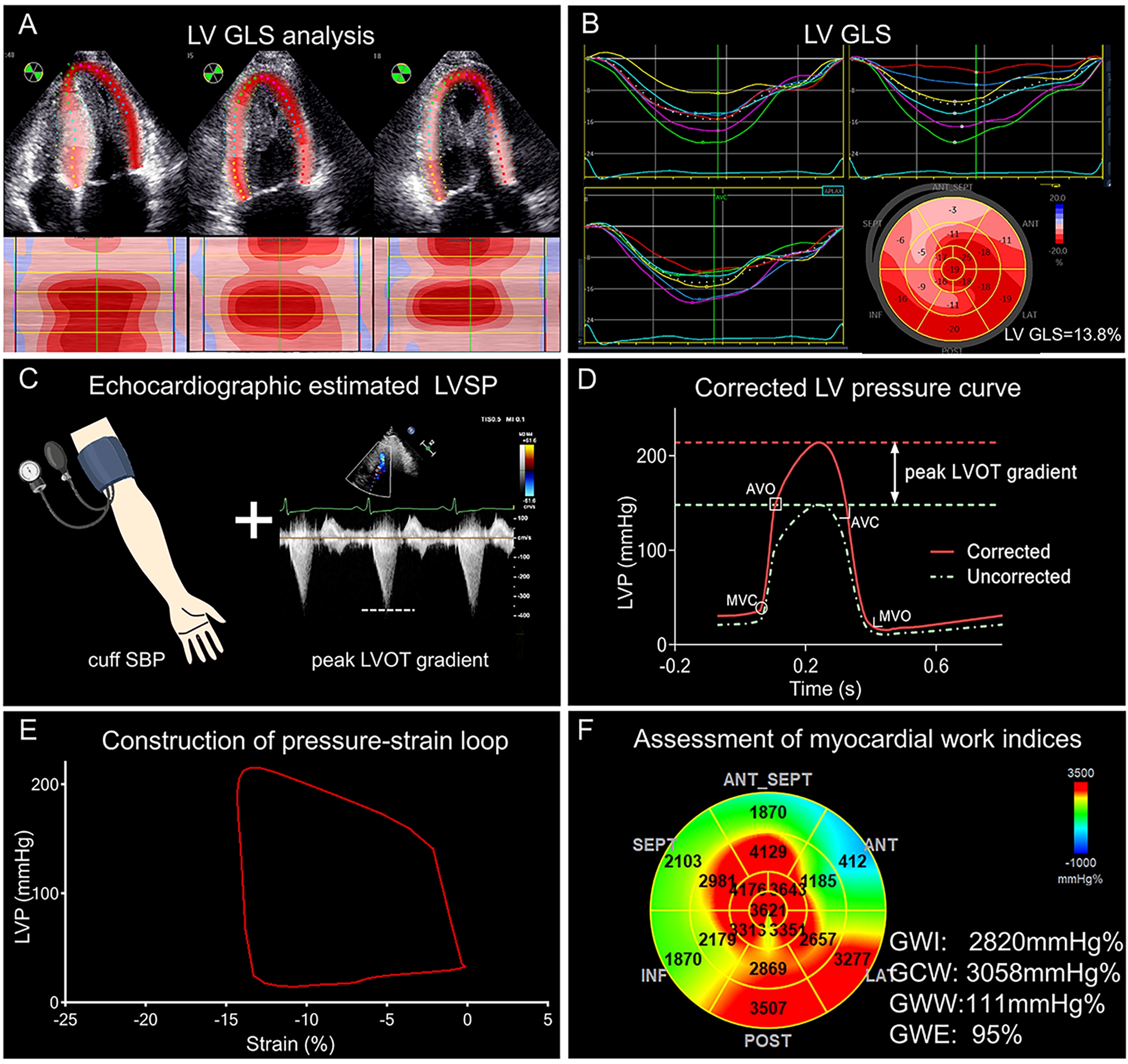


**Figure 1.** **Demonstration of the main steps in the assessment of myocardial work in HOCM.** (A-B) The LV GLS curve was analyzed with the standard apical two-, three- and four-chamber views by speckle tracking echocardiography. (C) LVSP was derived by adding the echocardiographic estimated peak LVOT gradient to the cuff SBP. (D) LV pressure curve was constructed by adjusting the standard LV pressure curve through identifying the valvular events and inputting the corrected LVSP. LV pressure-strain loops was constructed by combining LV pressure curve with LV GLS (E), and myocardial work indices were finally derived (F).

AVC= aortic valve closing; AVO= aortic valve opening; GCW= global constructive work; GLS= global longitudinal strain; GWE= global work efficiency; GWI= global work index; GWW= global wasted work; LV= left ventricle; LV= left ventricle pressure; LVOT= left ventricular outflow tract; LVSP= left ventricular systolic pressure; MVC= mitral valve closing; MVO= mitral valve opening; SBP= systolic blood pressure.


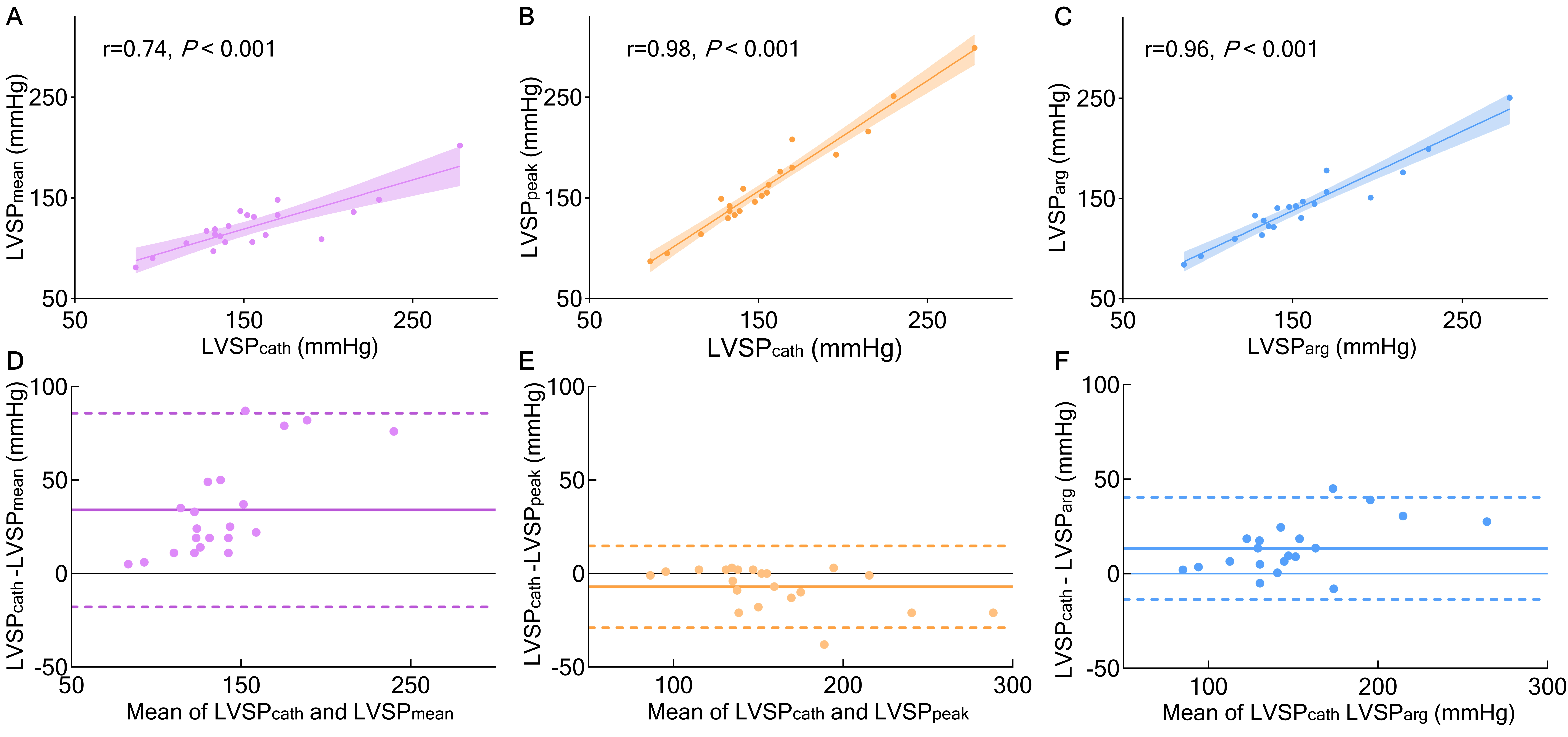


**Figure 2. Correlations and Bland-Altman plots of estimated LVSP and LVSP_cath_.** LVSP_peak_ (B and E; r=0.98, *P*<0.001; ICC=0.96, *P*<0.001; mean difference=-7.1 mmHg; 95% limits of agreement= -29.0 to 14.8 mmHg) has relatively higher correlation and consistency with LVSP_cath_ compared to LVSP_mean_ (A and D; r=0.74, *P*<0.001; ICC=0.51, *P*=0.060; mean difference=34.0 mmHg; 95% limits of agreement= -17.9 to 85.9 mmHg) and LVSP_arg_ (C and F; r=0.96, *P*<0.001; ICC=0.89, *P*=0.002; mean difference=13.5 mmHg; 95% limits of agreement= -13.6 to 40.5 mmHg).

ICC=intraclass correlation efficient; LVSP_cath_=invasively measured left ventricular systolic pressure; LVSP_peak_= the sum of the peak LVOT gradient and arterial systolic blood pressure; LVSP_mean_= the sum of the mean LVOT gradient and arterial SBP; LVSP_arg_= the sum of the arterial systolic blood pressure and the average of peak and mean LVOT gradients; Other abbreviations as in Figures 1.


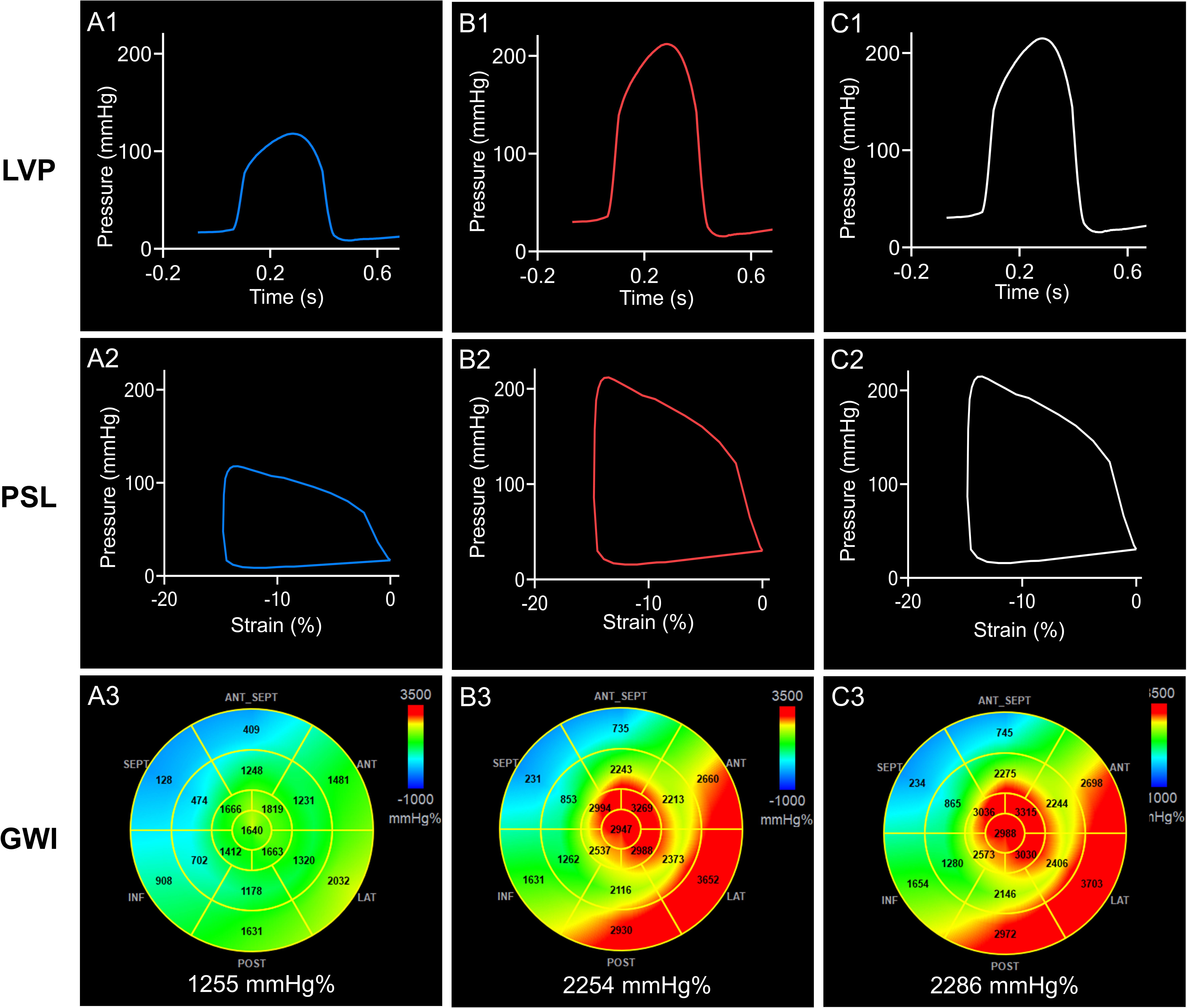


**Figure 3. Comparison of myocardial work analysis with different LVSP surrogate indices.** LVP curve, PSL, and myocardial work analysis results of a representative patient with cuff SBP (A1 – A3), the sum of cuff SBP and peak LVOT gradient (B1 – B3), or invasively measured LVSP (C1 – C3; setting as the gold standard) as the surrogate of LVSP. PSL= pressure-strain loop; Other abbreviations as in Figures 1.

**Reproducibility**


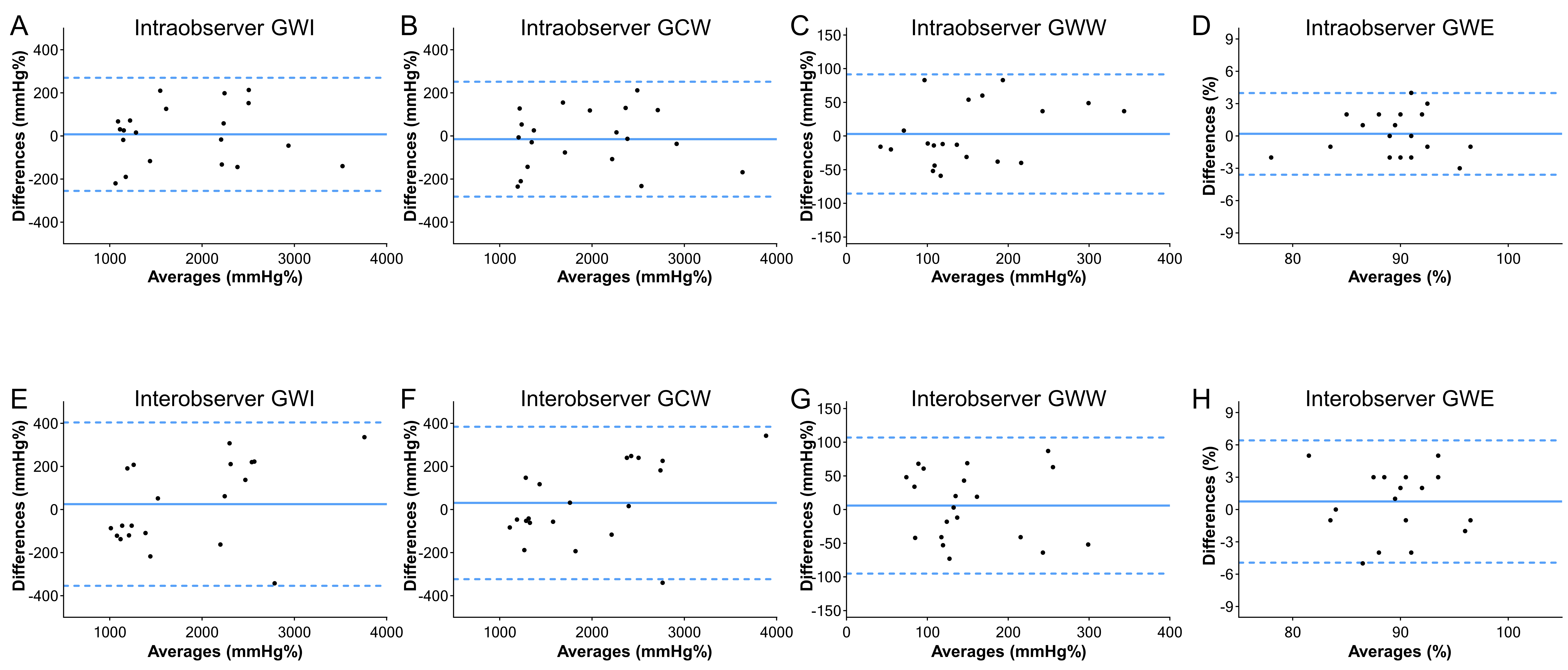


**Figure 4.** Bland-Altmann Plots of myocardial work indices. Intraobserver GWI, GCW, GWW, GWE(A-D) and interobserver GWI, GCW, GWW, GWE (E-H) variability of myocardial work indices as shown in Bland-Altmann Plots. Abbreviations as in Figures 1.

**Table 1. Intra- and Interobserver Reproducibility of Myocardial Work Parameters**

|  | **Intraobserver** | | |  | **Interobserver** | | |
| --- | --- | --- | --- | --- | --- | --- | --- |
|  | **ICC** | **95%CI** | ***P* value** |  | **ICC** | **95%CI** | ***P* value** |
| GWI | 0.984 | 0.959~0.993 | <0.001 |  | 0.969 | 0.924~0.988 | <0.001 |
| GCW | 0.982 | 0.956~0.993 | <0.001 |  | 0.972 | 0.931~0.989 | <0.001 |
| GWW | 0.852 | 0.663~0.939 | <0.001 |  | 0.738 | 0.448~0.887 | <0.001 |
| GWE | 0.897 | 0.760~0.958 | <0.001 |  | 0.762 | 0.500~0.898 | <0.001 |

CI= confidence interval; ICC = intraclass correlation efficient; GCW= global constructive work; GWE= global work efficiency; GWI= global work index; GWW= global wasted work
